# Astragalus membranaceus and its monomers treat peritoneal fibrosis and related muscle atrophy through the AR/TGF-β1 pathway

**DOI:** 10.3389/fphar.2024.1418485

**Published:** 2024-08-22

**Authors:** Li Sheng, Jinyi Sun, Liyan Huang, Manshu Yu, Xiaohui Meng, Yun Shan, Huibo Dai, Funing Wang, Jun Shi, Meixiao Sheng

**Affiliations:** ^1^ Department of Nephrology, Affiliated Hospital of Nanjing University of Chinese Medicine, Nanjing, China; ^2^ First Clinic Medical School, Nanjing University of Chinese Medicine, Nanjing, China; ^3^ Medical Experimental Research Center, First Clinic Medical School, Nanjing University of Chinese Medicine, Nanjing, China; ^4^ School of Traditional Chinese Medicine, Nanjing University of Chinese Medicine, Nanjing, China

**Keywords:** astragalus membranaceus, calycosin, peritoneal fibrosis, mesothelial mesenchymal transition, muscle atrophy, AR/TGF-β1 signaling pathway, mRNA sequencing, network pharmacology

## Abstract

**Background:** To anticipate the potential molecular mechanism of Astragalus membranaceus (AM) and its monomer, Calycosin, against peritoneal fibrosis (PF) and related muscle atrophy using mRNA-seq, network pharmacology, and serum pharmacochemistry.

**Methods:** Animal tissues were examined to evaluate a CKD-PF mice model construction. mRNA sequencing was performed to find differential targets. The core target genes of AM against PF were screened through network pharmacology analysis, and CKD-PF mice models were given high- and low-dose AM to verify common genes. Serum pharmacochemistry was conducted to clarify which components of AM can enter the blood circulation, and the selected monomer was further validated through cell experiments for the effect on PF and mesothelial mesenchymal transition (MMT) of peritoneal mesothelial cells (PMCs).

**Results:** The CKD-PF mice models were successfully constructed. A total of 31,184 genes were detected in the blank and CKD-PF groups, and 228 transcription factors had significant differences between the groups. Combined with network pharmacology analysis, a total of 228 AM-PF-related targets were identified. Androgen receptor (AR) was the remarkable transcription factor involved in regulating transforming growth factor-β1 (TGF-β1). AM may be involved in regulating the AR/TGF-β1 signaling pathway and may alleviate peritoneal dialysis-related fibrosis and muscle atrophy in CKD-PF mice. In 3% peritoneal dialysis solution-stimulated HMrSV5 cells, AR expression levels were dramatically reduced, whereas TGF-β1/p-smads expression levels were considerably increased.

**Conclusion:** AM could ameliorate PF and related muscle atrophy via the co-target AR and modulated AR/TGF-β1 pathway. Calycosin, a monomer of AM, could partially reverse PMC MMT via the AR/TGF-β1/smads pathway. This study explored the traditional Chinese medicine theory of “same treatment for different diseases,” and supplied the pharmacological evidence of “AM can treat flaccidity syndrome.”

## 1 Introduction

Chronic kidney disease (CKD) is a major disease affecting human health worldwide, and approximately 1.8% of CKD patients are classified as stage 4–5 in China (Wang et al., 2023), some of which inevitably enter the renal replacement therapy (RRT) process. Peritoneal dialysis (PD) is a valuable RRT, with advantages such as being able to be performed at home, better early survival, less impact on hemodynamics, better protection of residual renal function, and better clearance of medium macromolecular toxins (Bello et al., 2022).

PD has been recommended as the preferred treatment modality in many regions, especially during the COVID-19 pandemic (Kanjanabuch and Pongpirul, 2022; Nerbass et al., 2023; Sunnyraj et al., 2023). However, as patients are dialyzed for longer periods, the peritoneal structure and function change under the influence of many factors, such as touch and mechanical irritation of catheters, as well as peritonitis, so the pathological process of peritoneal fibrosis (PF) is difficult to avoid, which seriously restricts the long-term PD treatment of patients. Meanwhile, long-term PD is naturally complicated by related diseases, such as malnutrition, leading to muscle atrophy and even sarcopenia, and may negatively affect the quality of life of dialysis patients.

Mesothelial mesenchymal transition (MMT) of peritoneal mesothelial cells (PMCs), peritoneal inflammation (Rizzi et al., 2018; Szeto and Li, 2019; Perl et al., 2020), peritoneal angiogenesis (Huang et al., 2022; Masola et al., 2022), and epigenetic modifications (Bontempi et al., 2022; Shi et al., 2022) are widely studied mechanisms of PF. The peritoneum is composed of monolayer mesothelial cells, which act as the first line of defense against exposure to peritoneal dialysate. PMCs are damaged in the early pathological process of PF and gain strong invasiveness and induce accumulation of the extracellular matrix (ECM) by MMT, resulting in thickening of the connective tissue under the mesothelium. MMT is an early and reversible pathological process of PF, and TGF-β1 is a recognized fibrogenic factor therein (Shentu et al., 2021; Zhang et al., 2022).

Androgen receptor (AR) is one of the steroid hormone receptors which are essential transcription factors for mammalian physiology (Knerr et al., 2023) and are involved in the processes of cancer, reproductive system diseases, and musculoskeletal diseases (Guo et al., 2021; Efimenko et al., 2022; Pala et al., 2022). Recently, AR has also been shown to play an important role in fibrotic diseases, such as cardiac fibrosis (Tao et al., 2020; Shi et al., 2021), prostatic fibrosis (Kim et al., 2021), and renal fibrosis (Sun et al., 2018), and may be implicated in TGF-β1-regulated MMT. Furthermore, the property of AR modulates the expression of TGF-β1 (Yoon et al., 2006; Qi et al., 2008; Zhu et al., 2008; Nataf et al., 2019).

Astragalus membranaceus (AM) is a traditional Chinese herb with the role of tonifying Qi and has been widely used in multiple diseases. Chinese medicine theory analyzes that the pathogenesis of peritoneal fibrosis in CKD peritoneal dialysis patients is spleen and kidney qi deficiency. AM enters the spleen channel, tonifies qi and strengthens the spleen, disinhibit water and disperse swelling, and is mainly used to support positive qi, and removes the water evil, which is therapeutic for peritoneal dialysis. Furthermore, AM protects peritoneal function and its monomers can alleviate PF via multiple pathways (Li et al., 2014; Zhang et al., 2015b; Yu et al., 2018; Zhao J. Y. et al., 2020; Li et al., 2022). Calycosin, one of the total flavonoids of AM, has an antioxidant effect, promotes angiogenesis, and is anti-allergenic. Calycosin also plays an important anti-fibrotic role in renal fibrosis (Hu et al., 2023), liver fibrosis (Wang et al., 2022), and pulmonary fibrosis (Liu et al., 2022). We, therefore, tried to clarify the role of Calycosin in PF via the AR/TGF-β1 signaling pathway through RNA sequencing, network pharmacology, and animal and cell experiments.

## 2 Materials and methods

### 2.1 Cell cultures

Human PMCs (HMrSV5) were procured from JennioBiotech Co., Ltd. (Guangzhou, China). The cells were cultured at 37°C in a 5% CO2 incubator, using RPMI1640 medium (11875093, ThermoFisher Scientific, Rockville, MD) supplemented with 10% fetal bovine serum (ThermoFisher Scientific) and 1% penicillin/streptomycin (Invitrogen), and the incubator was maintained at 95% air atmosphere.

### 2.2 Cell viability assay

The HMrSV5 cells were counted and inoculated at 5 × 103/well in a 96-well plate and incubated for 24 h at 37°C in a 5% CO_2_ incubator. Then, the cells were treated with varying concentrations of Calycosin (HPLC ≥98%; 0, 80, 160, 320, 480, and 640 µM) and DHT (HPLC ≥98%; 0, 0.001, 0.01, 0.1, 1, and 5 µM) for 24 h. Cell proliferation was assessed using the Cell Counting Kit-8 (CCK-8; A311-02, Vazyme, Nanjing, China), and absorbance (OD value) at 450 nm was measured using an enzyme labeling device (EXL808, BIOTEK, Winooski, VT).

### 2.3 Animals

SPF wild-type C57BL/6 mice, aged 6–8 weeks and weighing 20–25 g, were obtained from Beijing Vital River Laboratory Animal Technology Co., Ltd. The mice were randomly housed in cages with six mice per group and provided with a full-price pellet diet and *ad libitum* access to drinking water. The room temperature was maintained at 18°C–22°C with a humidity level of 50%–60%. The mice were monitored daily for growth and overall health.

We first resected two-thirds of the left kidney after adaptive feeding and, on the seventh day thereafter, we excised the entire right kidney. After initial wound healing and restoration of vigor in mice to confirm no mortality, we intraperitoneally injected 4.25% peritoneal dialysis solution (PDS), and the injection volume was increased as the weight of the mice increased (1 mL/10 g). The dosage of AM was based on our previous experiments (Dai et al., 2024), at a low dose of 0.045 mg and a high dose of 0.090 mg AM per 10 g of body weight. After the intervention, the blood, peritoneum, and gastrocnemius muscles of the mice were collected and preserved. The animal experiments were approved by the Experimental Animal Ethics Committee of Nanjing University of Chinese Medicine (A220603) and carried out in accordance with the Guidelines for the Care and Use of Laboratory Animals (1985, NIH).

### 2.4 Serum enzyme-linked immunosorbent assay (ELISA)

The plasma was centrifuged at 3,000 rpm and 4°C for 20 min, and the levels of urea nitrogen and creatinine in the supernatant were detected by an automatic biochemical analyzer (BK-280, BIOBASE).

### 2.5 Masson staining

The peritoneal tissue was fixed in formalin and embedded in paraffin for slicing. Sections were sequentially stained with Régaud’s hematoxylin dye, Masson acidic fuchsin solution, and aniline blue aqueous solution. Masson staining was used to evaluate the pathological changes in the peritoneal tissues.

### 2.6 Immunofluorescence (IF) staining

The diaphragms of the mice were fixed, embedded, and dehydrated successively in a graded alcohol series. Tissue wax blocks were sliced for immunofluorescence double labeling staining, where green fluorescence labeled pan-cytokeratin indicated mesothelial cells and red labeled FN represented fibrosis. The staining aimed to assess the degree of fibrosis in the peritoneal mesothelium.

### 2.7 Immunohistochemistry (IHC) staining

The sections were blocked with BSA, stained with antibodies against FN overnight, and then incubated with the appropriate secondary antibody. After FN IHC staining, the microscopic images were fixed at the peritoneum. The number of FN positive cells was counted for statistical analysis.

### 2.8 Mesenteric RNA-seq difference analysis

Mesenteric RNA samples were extracted and checked by the NanoDrop™ One/OneC and Agilent 4,200 TapeStation system. After the sample assay was qualified, a library was built for mRNA capture and testing. The different libraries were then pooled according to the requirements of effective concentration and targeted offline data volume, and Illumina PE150 sequencing was performed.

### 2.9 Western blot

The total proteins from mouse peritoneal and gastrocnemius muscles and HMrSV5 were extracted on ice with RIPA buffer containing a mixture of phosphatase and protease inhibitors. The Pierce BCA Protein Assay Kit (Thermo Fisher Scientific) was utilized to determine the total protein concentrations. The proteins were separated using SDS-PAGE and then transferred onto a polyvinylidene fluoride (PVDF) membrane. Following the blocking step, the membrane was subjected to an overnight incubation at 4°C with the suitable primary antibody (diluted to 1:1,000): α-SMA (ab7817, Abcam), Vimentin (5741S, Cell Signaling Technology), E-cadherin (60,335, Proteintech), AR (19,672, Cell Signaling Technology), TGF-β1 (ab92486, Abcam), Smad2/3 (8,685, Cell Signaling Technology), p-Smad2/3 (8,828, Cell Signaling Technology), Myostatin (ab236511, Abcam), FBXO32 (ab168372, Abcam), TRIM63 (ab183094, Abcam), and β-actin (E4D9Z, Cell Signaling Technology). After being washed three times, the membranes were incubated with secondary HRP-linked rabbit/mouse IgG antibodies (diluted to 1:3,000, 7074S/7076S, Cell Signaling Technology), and subsequently visualized using Pierce ECL western blotting substrate (Thermo Fisher Scientific).

### 2.10 Network pharmacology analysis

Active ingredients and their corresponding potential targets of AM were screened from the TCMSP public database. The GeneCards, OMIM, and DisGent databases were used to obtain targets related to PF. The intersection targets of AM and PF were used as a potential target for the construction of compound-disease-target (C-D-T) and protein-protein interaction (PPI) networks. Gene ontology (GO) and Kyoto Encyclopedia of Genes and Genomes (KEGG) pathway enrichment analyses were performed based on intersection targets. A molecular docking analysis was performed to predict the binding of components in AM to the key gene AR.

### 2.11 Serum pharmacochemistry

AM was soaked in pure water for 30 min, and refluxed and extracted for 2 h, which was then repeated with the extract combined. The extract was concentrated under reduced pressure to 1 g/mL of raw medicinal material. The C57BL/6 mice were randomly divided into four groups of six mice as follows: 0, 1, 2 and 3 h. After 3 days of adaptive feeding, AM extract was orally administered at a dose of 10 g/kg per week. After the last administration, blood was collected using the retinal venous plexus blood collection method at varying time points. The plasma was centrifuged at 3,000 rpm and 4°C for 5 min, and the supernatant was removed and used for analysis according to a previous study (Kang et al., 2022).

### 2.12 Statistical analysis

Each mouse underwent at least three independent experiments. SPSS v12.0 software (SPSS, Chicago, IL) was employed to examine the data. The significance of the differences between the two groups was examined utilizing Student’s *t*-test. The data are presented as mean ± standard error, and a *P*-value <0.05 was regarded as statistically significant.

## 3 Results

### 3.1 Construction of a CKD-PF mouse model

**FIGURE 1:**
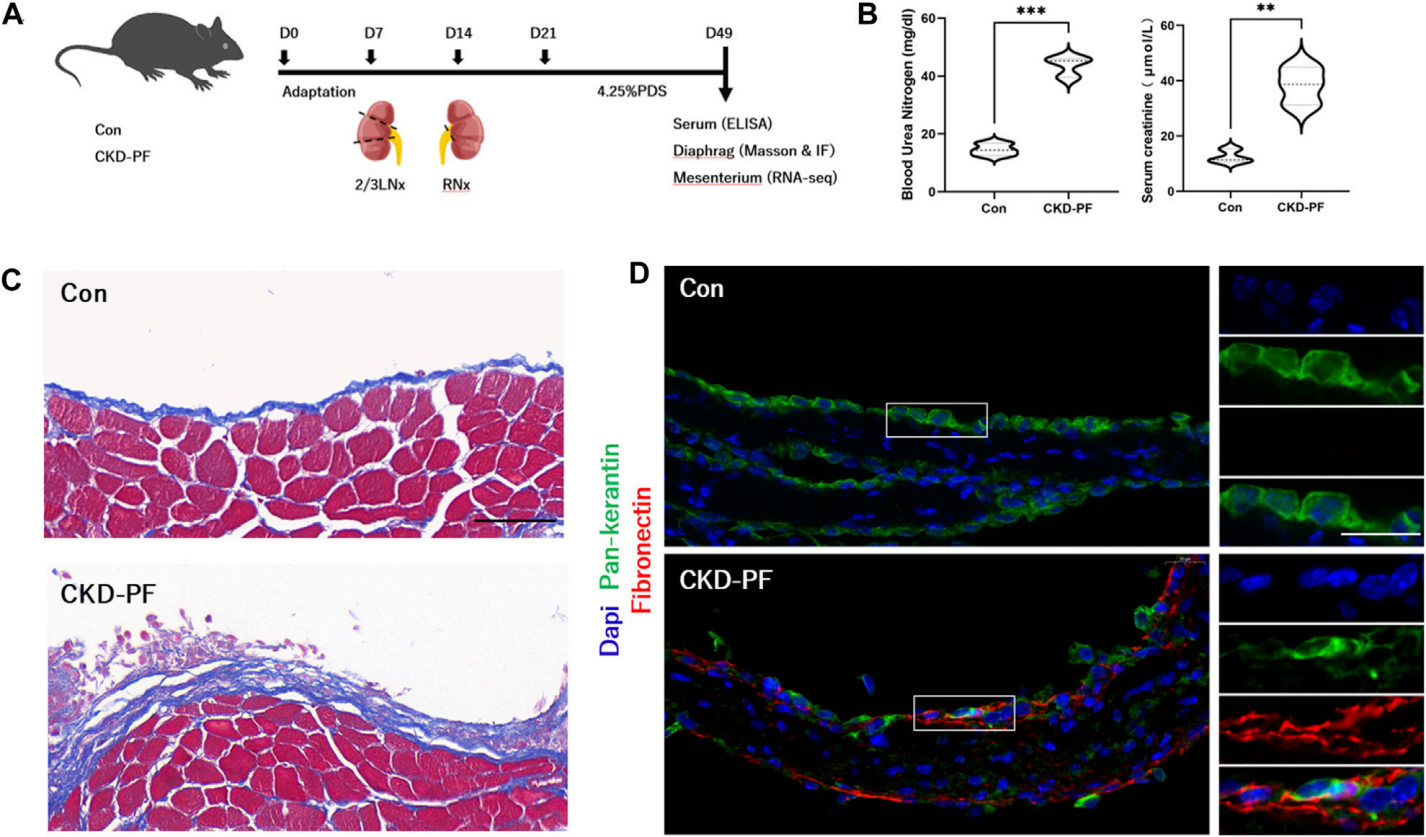
Construction of CKD-PF mice models. **(A)** Flow chart of the animal experiment. **(B)** Detection of mice renal function by ELISA. The data are expressed as mean ± SEM; **P < 0.01, ***P < 0.001 vs. the CKD-PF group, Student’s *t*-test. **(C)** Masson staining was utilized to detect pathological changes in the diaphragms of mice. Scale bar: 50 μm. **(D)** Immunofluorescence staining showing the FN expression in the diaphragms of mice. Scale bar: 20 μm. Blue (Dapi, nucleus), Green (Pan-keratin, mesothelial cells), Red (FN, fibrosis).

We examined animal tissues to evaluate the construction of the CKD-PF model (Figure 1A). Compared with the mice in the blank group, the blood urea nitrogen and creatinine of the mice in the model group appeared significantly elevated (Figure 1B), suggesting that the renal functions of the model mice were reduced. Subsequently, we examined mice diaphragms by Masson staining. As shown in Figure 1C, the peritoneal layer was thickened and the deposits of collagen under the peritoneum was increased in the model group mice. IF staining was used to estimate the level of FN expression in the peritoneum. Similarly, FN expressed in the model mice group was obviously higher than in the blank group (Figure 1D). Therefore, the CKD-PF mice models were successfully constructed.

### 3.2 AR may play an anti-PF role in CKD-PF mice by regulating the TGF-β1/smads signaling pathway

**FIGURE 2:**
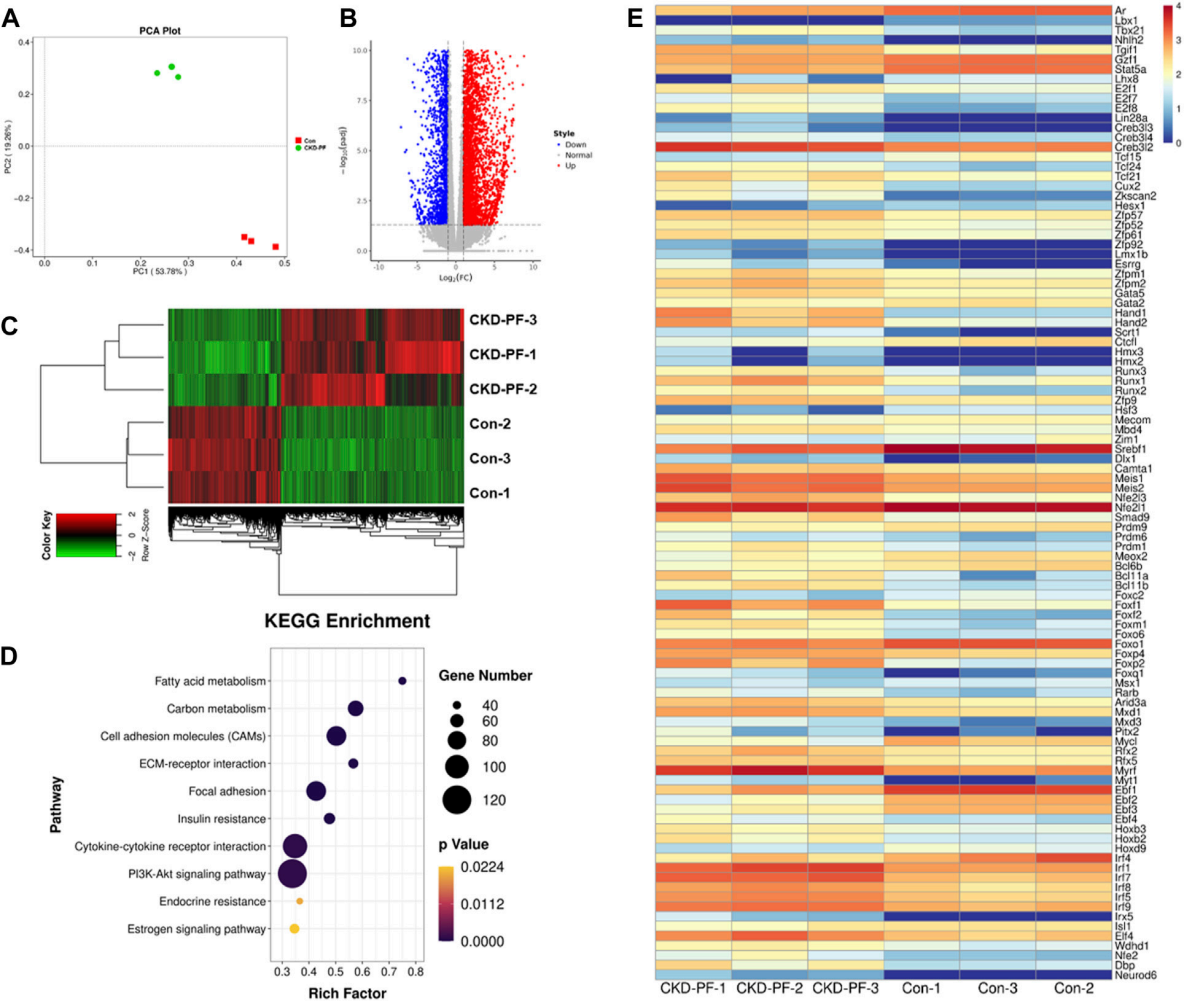
mRNA sequencing of mice mesenterium. **(A)** PCA Plot. Red (Con), Green (CKD-PF). **(B,C)** Differential genes detected in the blank and CKD-PF groups. **(D)** KEGG analysis of differential genes. **(E)** Transcription factors with significant differences between the two groups (*p* < 0.05)

To further explore the mechanism of PF, we performed mRNA sequencing using mice mesenterium from the blank and CKD-PF group mice. As shown in the PCA plot (Figure 2A), the degree of dispersion between the mice in the blank or CKD-PF groups was low, indicating that the mesenterium samples were similar in the respective groups. The degree of dispersion between the two groups was large, indicating that the mRNA expression between the groups was obviously different. A total of 31,184 genes were detected in the blank and CKD-PF groups, including 5,691 differential genes, of which 2,333 were decreased in the CKD-PF group (Figures 2B, C). Through the KEGG analysis of the differential genes, we found several biological process related to PF, such as ECM receptor interaction, focal adhesion, and the PI3K-Akt signaling pathway (Figure 2D), which were explored and verified to be regulated by TGF-β1/smads signaling in our previous study (Zhao J. et al., 2020; Dai et al., 2024).

Next, we explored the factors that may affect the expression of TGF-β1. Different from our past exploration, we focused on transcription factors. A total of 228 transcription factors had significant differences between the two groups, among which 70 were decreased in the CKD-PF group. Combined with the evaluation of expression and significance, AR was the remarkable transcription factor (Figure 2E), which is involved in regulating TGF-β1 (Xiao et al., 2022; Zhou et al., 2022). Therefore, we speculated that AR may play an anti-PF role in CKD-PF mice by regulating TGF-β1/smads signaling.

### 3.3 AM alleviates PF by modulating the AR/TGF-β1/smads pathway in CKD-PF mice

**FIGURE 3:**
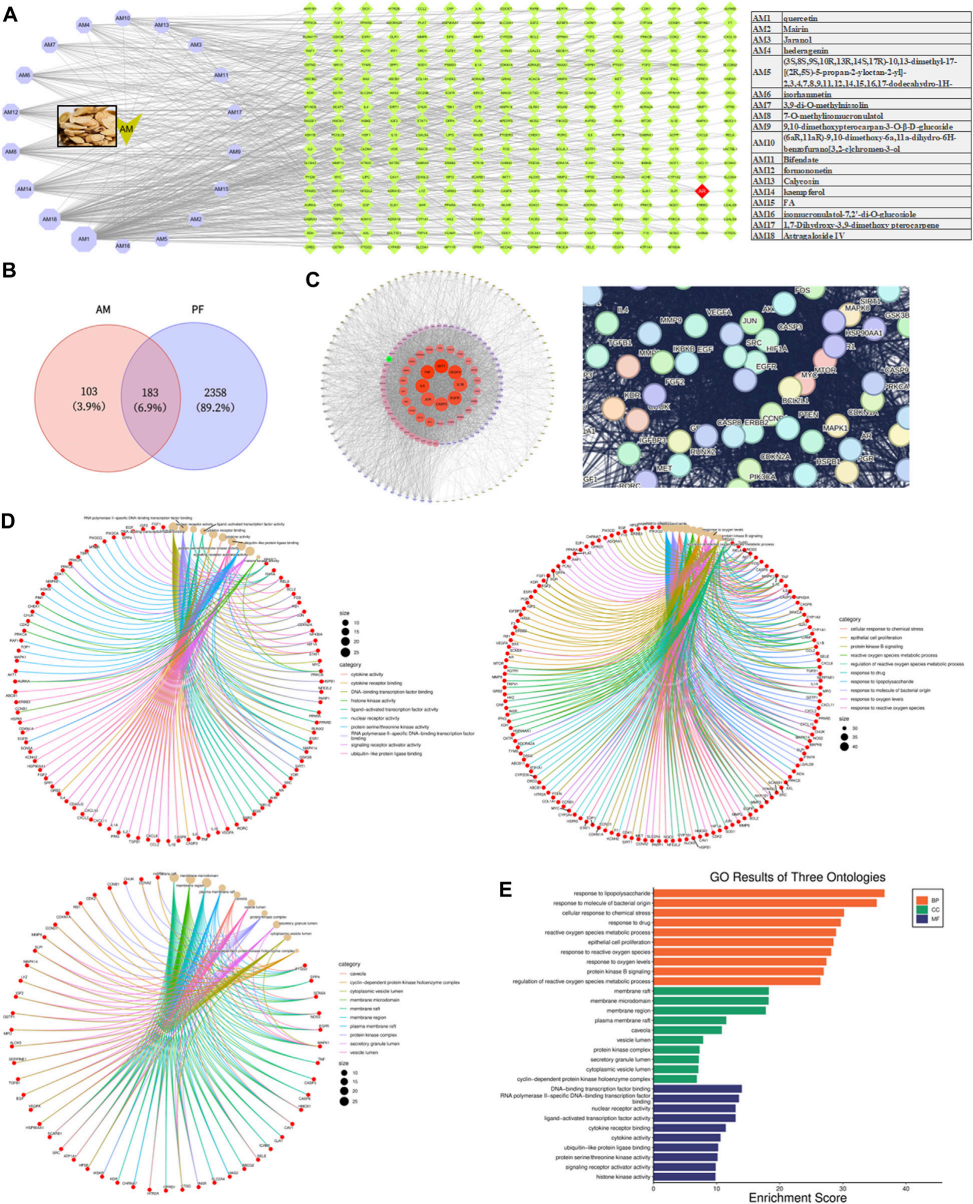
Network pharmacology analysis of AM and PF. **(A)** Components of AM and targets of these components. **(B)** Venn diagram showing common targets of AM and PF. **(C)** Top targets based on PPI network and interactions speculated by the String database. **(D,E)** GO analysis of common target between Calycosin and PF.

AM has been proven to alleviate PF (Gong et al., 2022). Through the TCMSP database, we found 17 components of AM that meet the standard, and added ASIV as per our previous study (Zhang et al., 2015a). Among the targets of these ingredients, we located AR (Figure 3A). Similarly, we discovered AR in the intersection target of AM and PF, which we speculated may interact with TGF-β1 by the String database (Figures 3B, C). The GO analysis showed the biological processes, cellular components, and molecular functions of common targets between Calycosin and PF (Figures 3D, E). Epithelial cell proliferation was related to MMT, the early pathological characteristics of PF. Therefore, we speculated that AM may lighten PF by modulating the AR/TGF-β1/smads pathway in CKD-PF mice.

**FIGURE 4:**
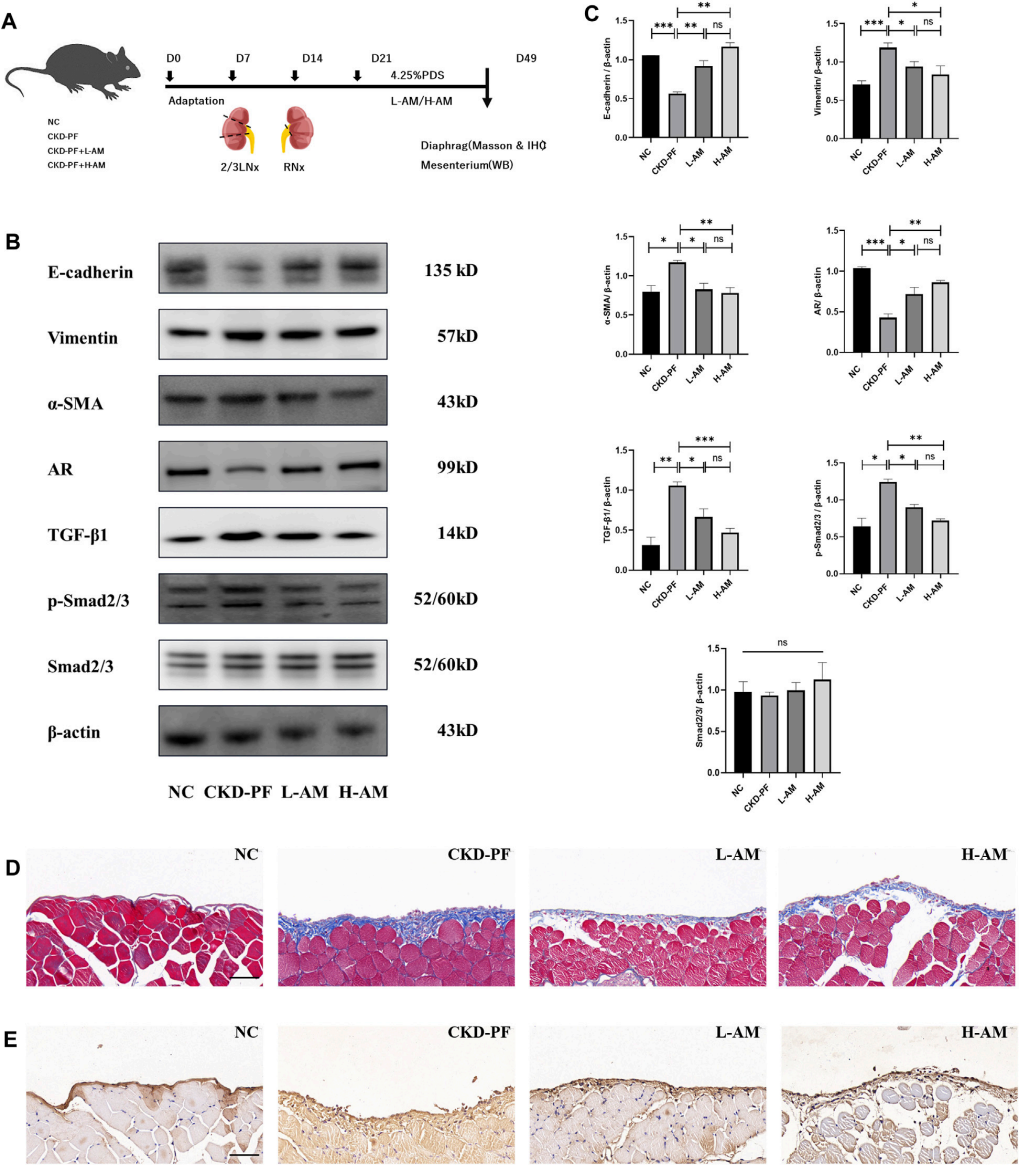
Effects of AM on PF. **(A)** Flow chart of animal experiment. **(B,C)** Expression of E-cadherin, Vimentin, α-SMA, AR, TGF-β1, p-Smad2/3, and Smad2/3 of the mesenterium in each group tested by western blot analysis. The data are expressed as mean ± SEM; *P < 0.05, **P < 0.01, ***P < 0.001 vs. the CKD-PF group, Student’s *t*-test. **(D)** Masson staining was utilized to detect pathological changes in the diaphragms of mice. Scale bar: 50 μm. **(E)** IHC staining was utilized to detect FN levels in the diaphragms of mice. Scale bar: 50 μm.

To verify this, mouse CKD-PF models were constructed again and were given high- and low-dose AM oral administration (Figure 4A). We tested the AR/TGF-β1/smads pathway and fibrosis indicators via collected diaphragms and mesenterium. As shown in Figures 4B, C, Vimentin and α-SMA were expressed higher in the model group compared to the NC group, while E-cadherin had the opposite trend, consistent with our previous animal experiments. High- and low-dose AM could ameliorate the above changes, and there was no statistical difference between these results. Similarly, Masson and IHC staining showed the same expression trend (Figures 4D, E).

### 3.4 AR may alleviate PD-related muscle atrophy by modulating TGF-β1

**FIGURE 5:**
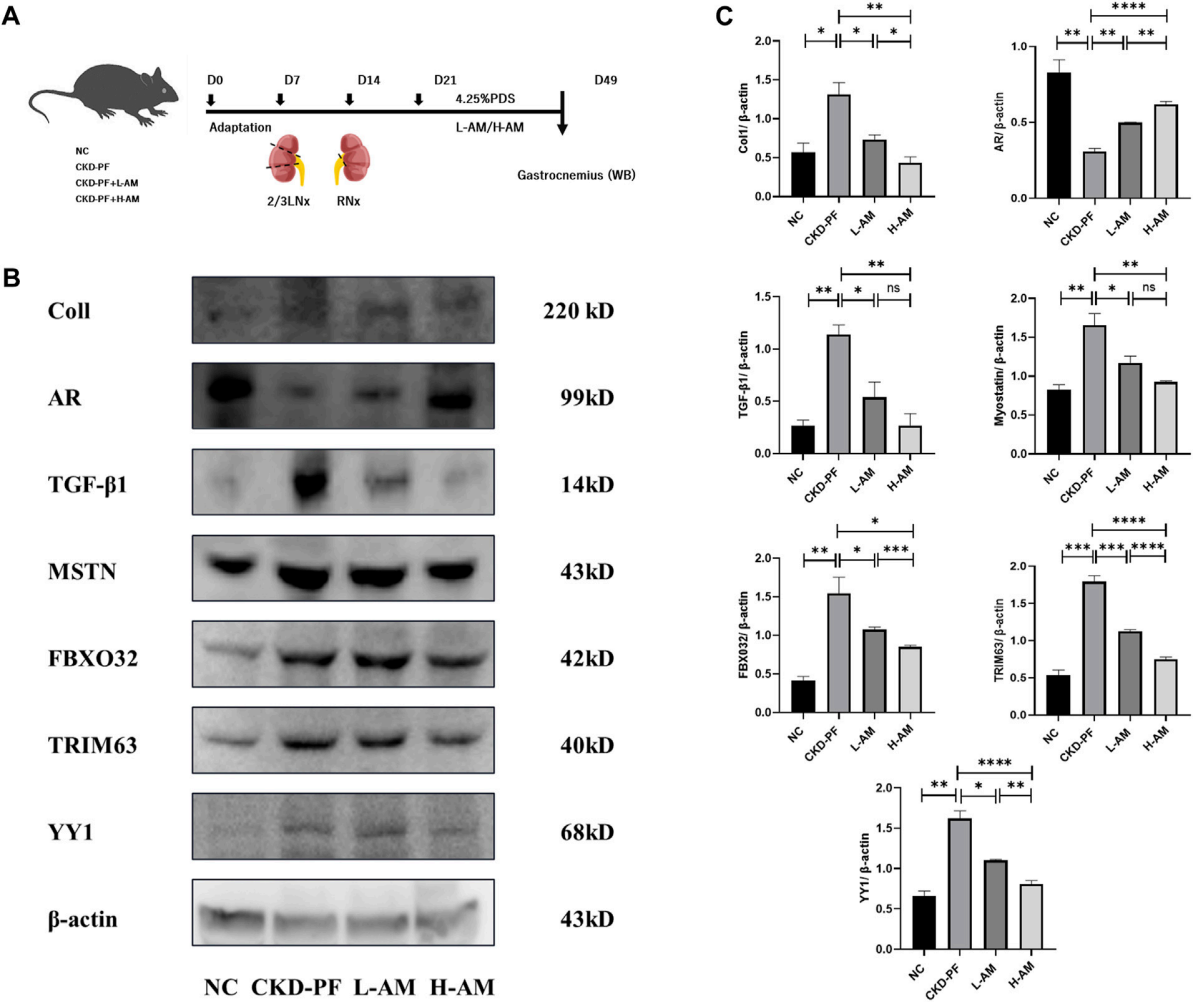
Effects of AM on muscle atrophy. **(A)** Flow chart of animal experiment. **(B,C)** Expression of Coll, AR, TGF-β1, MSTN, FBXO32, TRIM63, and YY1 of the gastrocnemius muscle in each group tested by western blot analysis. The data are expressed as mean ± SEM; *P < 0.05, **P < 0.01, ***P < 0.001, ****P < 0.001 vs. the CKD-PF group, Student’s *t*-test.

PF patients often experience muscle atrophy due to protein loss during the PD process. Dihydrotestosterone (DHT), a male hormone, stimulates AR secretion and has been widely used to treat amyotrophy. Therefore, we extirpated the gastrocnemius muscles of the same batch of mice to verify relevant indicators of muscle atrophy (Figure 5A). The muscle wasting index of gastrocnemius improved in the model mice, such as in Coll and MSTN, and may be associated with the degradation of muscle proteins indicators, like FBOX32, TRIM63, and YY1. AR was expressed lower and TGF-β1 expression was elevated, which was expected. A high dose of AM could partially reverse the indicators above and alleviate muscle atrophy, while low doses of AM had no effect (Figures 5B, C).

### 3.5 Calycosin may reduce PF

**FIGURE 6:**
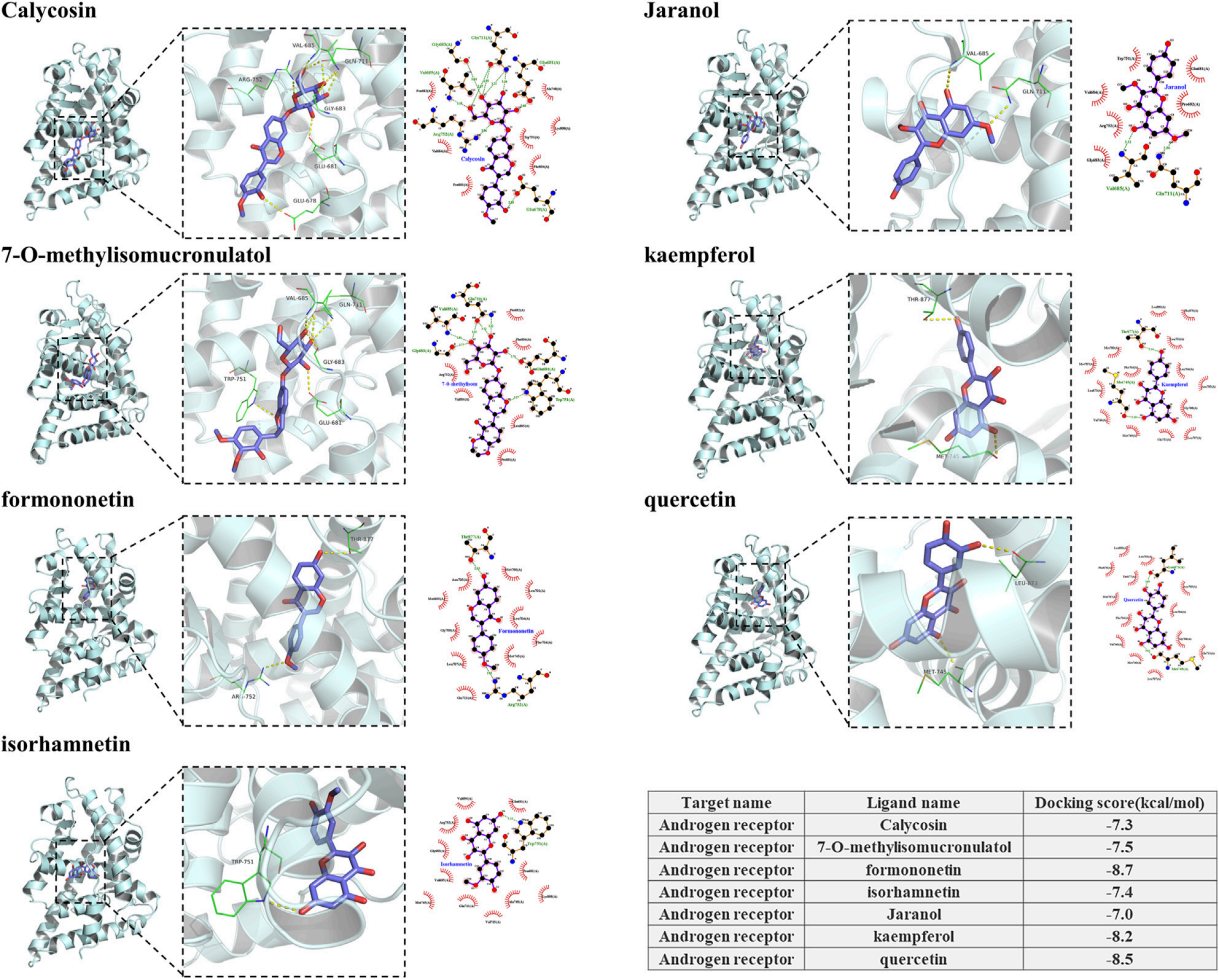
Components in AM that can dock with AR.

**FIGURE 7:**
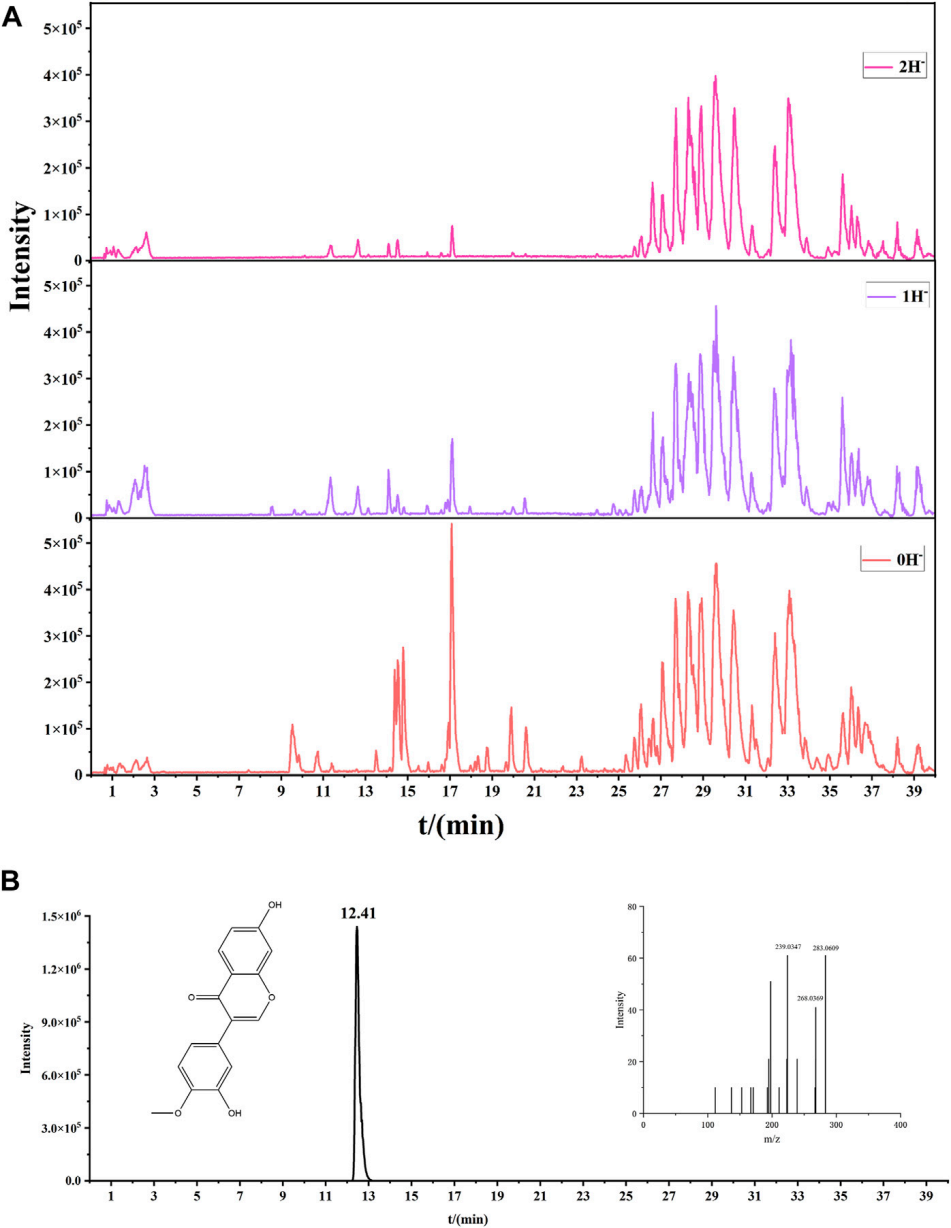
Serum pharmacochemistry of AM. **(A)** The base peak chromatogram of AM in the negative model at 2 h. **(B)** Calycosin in negative model at 2 h.

Through the verification of mice mesenterium tissue, we proved that AM lightened PF by modulating the AR/TGF-β1/smads pathway. We docked 18 components in Figure 3A with AR to find monomers that may combine with AR to produce efficacy. Seven components were found to combine with AR, including Calycosin, Jaranol, and kaempferol. All had cohesive energies lower than −7 kcal/mol, suggesting a stable docking outcome (Figure 6). To further clarify which components of AM can enter the blood circulation and thus play an effective role, we conducted serum pharmacochemistry. As shown in Figure 7, the presence of Calycosin began 2 h after administration. Therefore, we further speculated that Calycosin, the effective ingredient of AM, may effectively reduce PF via the AR/TGF-β1/smads pathway.

### 3.6 Calycosin relieves PMCs MMT through the AR-mediated TGF-β1/smads pathway

**FIGURE 8:**
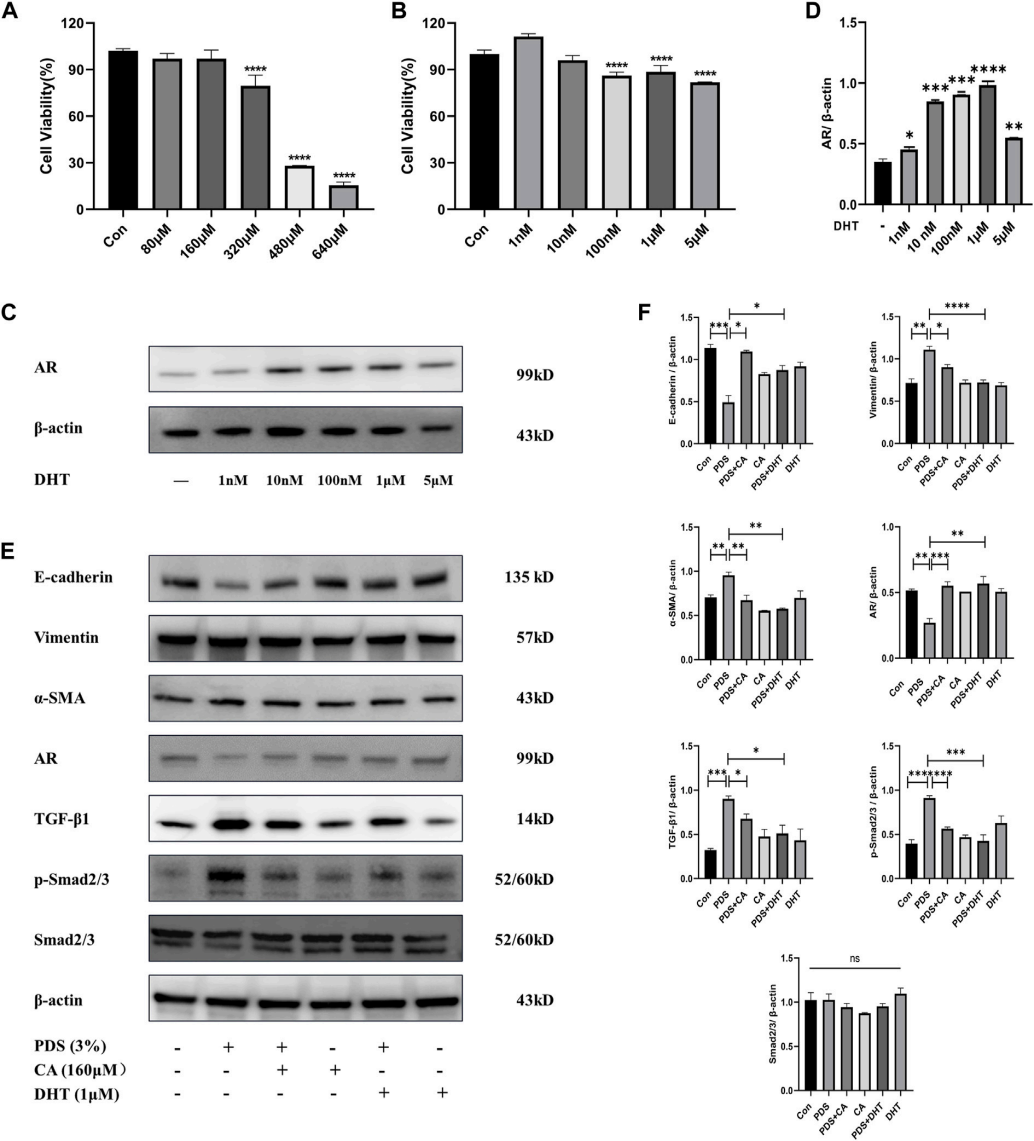
Effects of Calycosin on relieving PMC MMT through AR mediated TGF-β/smad pathway. **(A)** CCK8 assay to detect cell viability of different concentrations of Calycosin on PMCs. **(B)** CCK8 assay to detect cell viability of different concentrations of DHT on PMCs. The data are expressed as mean ± SEM; ****P < 0.0001 vs. the Con group, Student’s *t*-test. **(C–D)** AR expressions of PMCs intervened by different concentrations of DHT. The data are expressed as mean ± SEM; ****P < 0.0001 vs. the blank group, Student’s *t*-test. **(E–F)** Expression of E-cadherin, Vimentin, α-SMA, AR, TGF-β1, p-Smad2/3, and Smad2/3 of PMCs in each group tested by western blot analysis. The data are expressed as mean ± SEM; *P < 0.05, **P < 0.01, ***P < 0.001, ****P < 0.001 vs. the Con group, Student’s *t*-test.

To further investigate the mechanism of AR and the effective role of Calycosin in anti-PF, we conducted a cell experiment. Firstly, a CCK8 assay was used to detect cell viability of different concentrations of Calycosin on PMCs. As shown in Figure 8A, we chose 160 μM as the intervention concentration. Likewise, we tested cell viability of different concentrations of DHT on PMCs, the agonist of AR, and chose 1 μM as the intervention concentration (Figures 8B–D). Subsequently, western blotting was used to detect the effect of Calycosin and DHT on our target AR under PDS intervention, and the changes of fibrosis indicators and TGF-β1/Smads pathway. As shown in Figures 8E, F, 3% PDS could obviously raise the expression of Vimentin and α-SMA, and reduce the expression of E-cadherin. During this process, the expression of AR decreased, and the TGF-β1/smads pathway was activated. Calycosin could partially ameliorate the above changes. Next, we selected DHT to clarify the role of AR, which served as a positive control drug to expound the pesticide effect of Calycosin. The results suggested that Calycosin could lighten PF by increasing AR and reducing the expression of the TGF-β1/Smads pathway.

## 4 Discussion

MMT is an important mechanism in fibrotic diseases, including pulmonary fibrosis, kidney fibrosis, hepatic fibrosis, and tuberculous pleural fibrosis (Hsieh et al., 2019; Chen et al., 2020; Liu et al., 2020; Shi et al., 2023). MMT, as an important reversible segment in PF (Masola et al., 2022), has been widely studied and explored. Activated protein C could ameliorate PF by reducing the expression of Vimentin and α-SMA (markers of MMT) (Giri et al., 2023). Histone deacetylases (HDACs), such as HDAC1 (Bontempi et al., 2022) and HDAC8 (Zhou et al., 2023), were also found to improve PF by reversing MMT. In traditional Chinese medicine (TCM) studies, many herbs and their monomers have been found to be effective in reversing MMT; for instance, parthenolide (Zhang et al., 2022). We previously found that Astragaloside IV, the effective component of AM, and Asiaticoside (Sun et al., 2023), the effective monomer of *Centella asiatica*, could effectively reduce MMT and improve PF.

There are many options to construct PF mice models. We formerly used intraperitoneal injections of chlorhexidine gluconate or high sugar PDS, and both can successfully form model mice. To simulate the body state of PF more realistically under renal failure conditions, a 5/6 nephrectomy combined with PDS daily injection was used to effectively construct CKD-PF mice models. MRNA sequencing was performed to search for novel targets of PF. Our previous studies focused on differential genes or enriched pathways. However, AR, a distinct transcription factor, attracted our attention in this study. Firstly, AR expression was high in the mesentery of mice in both the blank and model groups, and the difference between the two groups was extremely obvious. In addition, AR has been found to inhibit the expression of TGF-β1, which is universally acknowledged as a fibrogenic factor and a inducer of MMT (Peng et al., 2022). Lastly, but also importantly, CKD patients with long-term PD often experience muscle atrophy along with PF due to protein loss, as mentioned (Bataille et al., 2021). AR can regulate skeletal muscle mass by regulating protein synthesis (Forouhan et al., 2022). TGF-β1, which is regulated by AR, can also negatively affect skeletal muscle (Ruan et al., 2022; Dai et al., 2023). Therefore, we speculated that AR and regulated TGF-β1 may be the co-targets of PF and muscle atrophy.

AM can effectively ameliorate PF. By network pharmacology analysis of AM and PF, we found AR among the common targets. We performed further validation by constructing CKD-PF mice models and administrating high and low doses of AM. The results suggested that both doses of AM were able to ameliorate the expression of fibrotic indicators in the mesenterium of model mice by modulating the AR/TGF-β1/smads pathway, prompting a therapeutic effect on PF. However, only high-dose AM was able to improve gastrocnemius muscle atrophy in the model mice, with Coll, MSTN, and YY1 being phenotypes of skeletal muscle atrophy; FBXO32 and TRIM63 were indicators of protein synthesis. This may be related to the traditional Chinese medical theory that large doses of AM can treat flaccidity syndrome (muscle atrophy).

We focused on investigating the pharmacological effects of TCM components on PF, and we discovered that some monomers may be alternative choices, depending on their advantages such as smaller molecular weights and stronger stability, based on experiments *in vitro* and *in vivo*. Here, we first performed molecular docking between the components of AM and the target protein AR, and found that seven monomers could stably bind to AR, including Calycosin. Subsequently, we performed TCM serum medicinal chemistry to clarify which components of AM have the ability to be incorporated into the blood and then exert their effects, and Calycosin was, again, involved. Therefore, we finally chose Calycosin for the next step to verify the cellular mechanism.

In the cell experiments, we used 3% PDS as the modeling drug according to previous experience, 160 μM Calycosin as the therapeutic drug concentration, and 1 μM DHT as the positive drug concentration based on CCK8, to investigate the mechanism of antagonizing HMrSV5 MMT. The expression of AR was downregulated and the fibrogenic indicators were upregulated after modeling, while Calycosin was able to partially reverse these alterations, which is consistent with DHT. Unfortunately, our study did not validate the suppressive effect of AR on the TGF-β1/smads pathway *in vivo* using DHT as a positive drug. We will follow up with further experiments to verify the mechanism above and explore the detailed regulatory mechanisms. Despite its preliminary character, this study can clearly indicate that AR could inhibit the TGF-β1/smads pathway, and Calycosin was able to increase the expression of AR and decrease the TGF-β1/smads pathway, resulting in the reverse of PMC MMT.

We found that AM is effective in treating both PF and muscle atrophy, which proved the TCM theory of “same treatment for different diseases,” to some extent. In PF, we further found that Calycosin, the monomer of AM, could partially reverse PMC MMT via the AR/TGF-β1/smads pathway. However, we did not delve into the mechanism of AM in ameliorating muscle atrophy, and we will further verify this in subsequent experiments.

## 5 Conclusion

**FIGURE 9:**
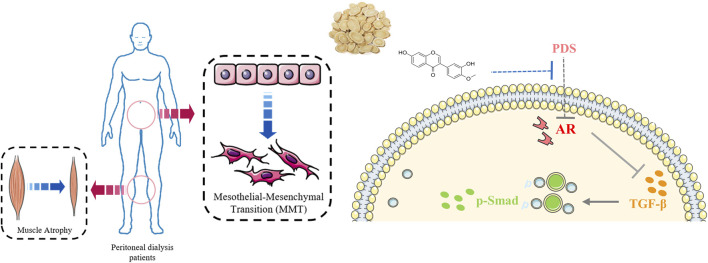
Brief mechanism diagram of this study.

Collectively, our study verified the effect of AM on ameliorating PF and related muscle atrophy via the co-target AR and modulated AR/TGF-β1 pathway (Figure 9). Combined with serum medicinal chemistry, network pharmacology, and molecular docking, we determined that Calycosin could partially reverse PMC MMT via the AR/TGF-β1/smads pathway. This study helps to clarify the detailed pharmacological mechanism of AM on PF and muscle atrophy, and not only explored the TCM theory of “same treatment for different diseases,” but also supplied the pharmacological evidence of “AM can treat flaccidity syndrome.”

## Data Availability

The original contributions presented in the study are publicly available. This data can be found here: https://www.ncbi.nlm.nih.gov/bioproject/PRJNA1147478.
